# Mental health of rural doctors and influencing factors in Hebei, China

**DOI:** 10.1017/S1463423625100200

**Published:** 2025-07-11

**Authors:** Yatian Liu, Hanling Di, Yunqing Xu, Ziwei Yang, Ye Zhang, Yuqi Yuan, Ning Zhang, Jiajun Li, Biao Zhao, Yu Wang, Yujie Niu, Longmei Tang

**Affiliations:** 1 Department of Epidemiology and Health Statistics, School of Public Health, Hebei Medical University, Shijiazhuang, Hebei, China; 2 Shijiazhuang Center for Disease Control and Prevention, Shijiazhuang, Hebei, China; 3 Hebei Key Laboratory of Intractable Pathogens, Shijiazhuang Center for Disease Control and Prevention, Shijiazhuang, Hebei, China; 4 Department of Infection Management, Liyang Hospital of Traditional Chinese Medicine, Liyang, Jiangsu, China; 5 Rural Physician College, Hebei Medical University, Shijiazhuang, Hebei, China; 6 Hebei Provincial Government Service Center, Shijiazhuang, Hebei, China; 7 Department of Epidemiology and Endemic Disease Control, Chaoyang District Center for Disease Control and Prevention, Beijing, China; 8 Hebei Medical University, Shijiazhuang, Hebei, China; 9 Medical School of Nanjing University, Nanjing, Jiangsu, China; 10 Department of Hygiene Inspection, School of Public Health, Hebei Medical University, Shijiazhuang, Hebei, China; 11 Department of Occupational Health and Environmental Health, Hebei Medical University, Shijiazhuang, Hebei, China; 12 Hebei Key Laboratory of Environment and Human Health, Shijiazhuang, Hebei, China

**Keywords:** adults, health care, health psychology, quantitative methods, regression, risk factors

## Abstract

**Aim:**

This study investigated the factors influencing the mental health of rural doctors in Hebei Province, to provide a basis for improving the mental health of rural doctors and enhancing the level of primary health care.

**Background:**

The aim of this study was to understand the mental health of rural doctors in Hebei Province, identify the factors that influence it, and propose ways to improve their psychological status and the level of medical service of rural doctors.

**Methods:**

Rural doctors from 11 cities in Hebei Province were randomly selected, and their basic characteristics and mental health status were surveyed via a structured questionnaire and the Symptom Checklist-90 (SCL-90). The differences between the SCL-90 scores of rural doctors in Hebei Province and the Chinese population norm, as well as the proportion of doctors with mental health problems, were compared. Logistic regression was used to analyse the factors that affect the mental health of rural doctors.

**Results:**

A total of 2593 valid questionnaires were received. The results of the study revealed several findings: the younger the rural doctors, the greater the incidence of mental health problems (OR = 0.792); female rural doctors were more likely to experience mental health issues than their male counterparts (OR = 0.789); rural doctors with disabilities and chronic diseases faced a significantly greater risk of mental health problems compared to healthy rural doctors (OR = 2.268); rural doctors with longer working hours have a greater incidence of mental health problems; and rural doctors with higher education backgrounds have a higher prevalence of somatization (OR = 1.203).

**Conclusion:**

Rural doctors who are younger, male, have been in medical service longer, have a chronic illness or disability, and have a high degree of education are at greater risk of developing mental health problems. Attention should be given to the mental health of the rural doctor population to improve primary health care services.

## Introduction

China is the largest developing country in the world. China’s primary medical technology and medical development level play vital roles worldwide. In China, 50.32% of the population lives in rural areas (Liang and Lv, 2019). Rural doctors constitute an essential part of China’s medical and health team, accounting for 20.71% of health technicians (Chen *et al*., 2019). They are significant human resources that directly provide preventive health care and basic medical services for most farmers (Lin *et al*., 2014). In particular, since the beginning of the 21st century, the government has issued a series of policy documents related to rural doctors, who have become important providers of basic medical and public health services. They play a crucial role in the rural medical system and deliver health services to thousands of households (Liu, 2004; Zhang *et al*., 2019), which is a force that cannot be ignored in carrying out rural health work and promoting rural primary health care (Zhang and Yang, 2019). Given the severe shortage of health resources in rural areas, rural doctors play an important role in providing basic health manpower and basic health services to the rural population (Hu *et al*., 2017). The training and use of rural doctors who can ‘stay and work’ has solved many medical and health problems for rural residents. The health of rural doctors directly affects the quality of primary health care, the relationship between doctors and patients, and even the stability and development of rural society (Wu *et al*., 2010). Therefore, maintaining the health of rural doctors is an important guarantee for the implementation of various types of health work in rural areas.

In addition to physical health, attention to health conditions should also be given to mental health, as the two factors are closely interconnected (Happell *et al*., 2017). Mental health can be defined as the absence of mental disease or it can be defined as a state that also includes the biological, psychological, or social factors that contribute to an individual’s mental state and ability to function within the environment (Manwell *et al*., 2015). Mental health impacts individuals’ personal and social functioning (Dore and Caron, 2017).

With the development of the social economy, improvements in living standards, the advancement of new medical reform policies, and the enhancement of public awareness of medical care, higher requirements are put forward for medical quality and medical safety. Consequently, the psychological pressure and work intensity of medical staff are greater than those of other occupations. Moreover, tense doctor-patient relationships further increase the pressure on medical staff, making mental health problems increasingly prominent (Gazelle *et al*., 2015; Lv *et al*., 2018). Research shows that doctors report a higher rate of depression and anxiety compared to the general population (Pattani *et al*., 2001; Garelick *et al*., 2007), with a greater incidence of mental illness (Pappas *et al*., 2016; Harvey *et al*., 2021).

At present, investigations on doctors’ mental health in China have focused mainly on the top three hospitals, general hospitals and specialized hospitals (Wang *et al*., 2013; Lin *et al*., 2013; Lin *et al*., 2017). They rarely involve primary hospitals or community health service centres. There are few investigations and studies on rural doctors. The sample size is relatively small, and the research period is relatively long (Chen *et al*., 2009; Wu, 2011; Wang and Wang, 2014), which is insufficient for research on the influence on the mental health of rural doctors and its influencing factors in the new era and environment.

The geographical location of Hebei Province is very important. Surrounding Beijing, the political, economic, and cultural centre of China, it serves as a link between the Northeast Region and various provinces within Shanhaiguan, making its status highly representative. To clarify the mental health status of rural doctors in Hebei Province, this survey randomly selected rural doctors. The Symptom Checklist-90 (SCL-90) was used to evaluate their mental health status, while multifactor analysis identified the factors influencing it. This study provides a scientific basis for health administrations to formulate relevant policies, contributing to the improvement of rural doctors’ psychological condition and their ability to provide primary health care, ultimately fostering the development of the primary health care system.

## Methods

### Setting

The survey subjects are rural doctors in 11 prefecture-level cities, including Shijiazhuang, Tangshan, Qinhuangdao, Handan, Xingtai, Baoding, Zhangjiakou, Chengde, Cangzhou, Langfang, and Hengshui.

### Sample size estimation

The sample size was estimated according to the sample size formula for estimating finite population proportion. Based on the total number of rural doctors in Hebei Province (*N* = 83,659), with a significance level α set at 0.05, an allowable error *δ* of 1%, and population standard deviation (*S* = 0.25), the minimum required sample size was determined to be 2334. Taking into account the response rate and efficiency of the questionnaire, the final sample size was determined to be 2500.

### Study design

**Figure 1.**
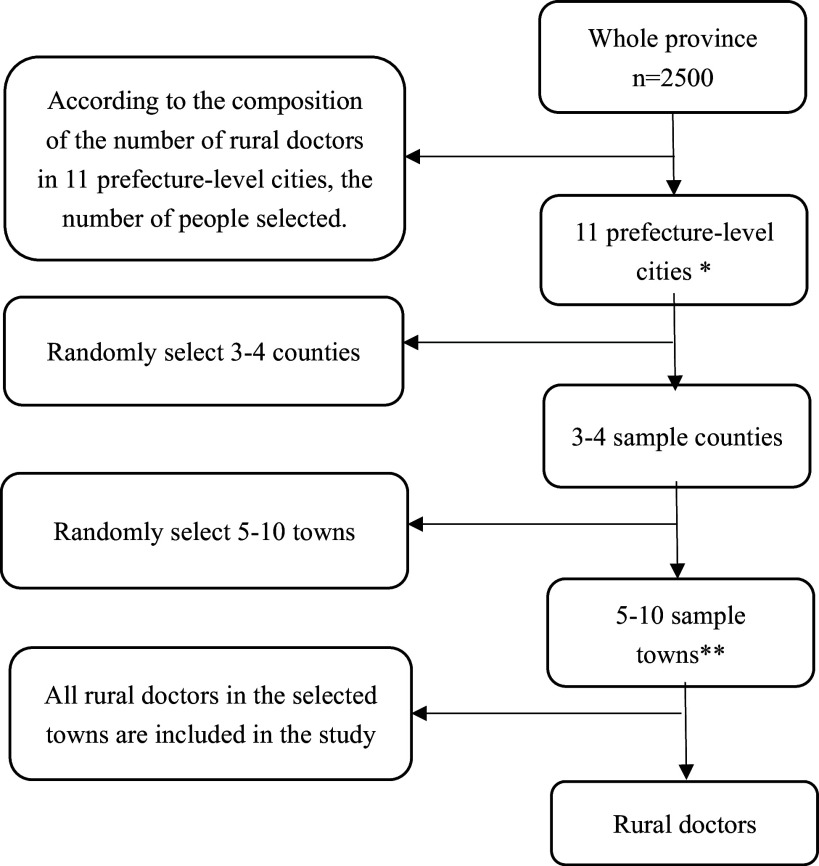
Sampling process flow chart. * Including two county-level cities, Xinji City (belonging to Shijiazhuang City) and Dingzhou City (belonging to Baoding City). ** According to the estimated number of sampled persons in each city, the number of selected townships varies

The multistage stratified cluster sampling method is used to extract research objects. According to the composition of the number of rural doctors in 11 prefecture-level cities, 3–4 counties (county-level cities) were selected from each city, 5–10 towns from each county (county-level cities), and all villages of each selected township (town) were included in the survey. The survey uses one rural doctor from each village clinic to complete a questionnaire, as shown in Figure 1.

### Measures

The survey uses a structured questionnaire, which consists of a self-designed basic condition questionnaire and a Symptom Checklist-90 (SCL-90) for rural doctors. Each item uses a 5-level scoring method (none = 0, light degree = 1, moderate = 3, heavy = 4, severe = 5), which takes approximately 10–15 minutes to complete (Derogatis *et al*., 1976; Bech *et al*., 2014). Primary conditions include gender, age, education, income, work intensity, and physical health. In this study, we used the Chinese version of the SCL-90 to assess rural doctors’ mental disorders and mental health in Hebei Province. The Chinese version of the SCL-90 includes 90 items in 9 dimensions (excluding ‘others’). The main symptom dimensions evaluated are somatization (SOM), obsessive-compulsive disorder (OCS), interpersonal sensitivity (INTS), depression (DEPR), anxiety disorder (ANX), hostility (HOS), phobia (PHOA), paranoia thoughts (PARI), and psychosis (PSY) (Wang, 1984).

The Cronbach’s alpha coefficient of the questionnaire is 0.984. The KMO value is 0.988, and Bartlett’s test of sphericity is passed with a significance level of 0.05. These findings indicate that the questionnaire has good reliability and validity.

### Variables

According to the scoring standard of the SCL-90 scale, if the total score exceeds 160, or if the number of positive items exceeds 43, or if any factor score exceeds 2 points, the item should be considered positive; if the somatization dimension score is greater than or equal to 24 points, it is considered to indicate somatization symptoms, that is, there are mental health problems; if the obsessive‒compulsive score is greater than or equal to 20 points, it is considered to indicate obsessive‒compulsive symptoms; if the interpersonal relationship score is greater than or equal to 18 points, it is considered sensitive to interpersonal relationships; if the depression dimension score is greater than or equal to 26 points, it is considered to indicate depressive symptoms; if the anxiety dimension score is greater than or equal to 20 points, it is considered to indicate anxiety symptoms; if the hostile dimension score is greater than or equal to 12 points, it is considered to indicate hostile symptoms; and if the terror dimension score is greater than or equal to 14 points, it is considered to indicate horror symptoms; if the paranoid dimension score is greater than or equal to 12 points, it is considered to indicate paranoid symptoms; and if the psychotic dimension score is greater than or equal to 20 points, it is considered to indicate psychotic symptoms.

The participants were classified into four groups according to age: ≤40, 41–50, 51–60, and > 60 years. The education status is divided into four groups, namely no medical education, technical secondary school, junior college, and undergraduate. The working times of the participants were classified into four groups: 1–3, 4–7, 8–11, and ≥12 hours.

### Statistical analysis

The data were analysed via the Statistical Package for Social Scientists (IBM SPSS version 25.0). The statistical frequency and proportion were used to describe the sample characteristics. The Chi-square test was also used to compare the proportion of positive screenings among rural doctors with different sociodemographic characteristics. Moreover, t-tests were used to compare the average SCL-90 scores (each of the nine dimensions) between the general Chinese population and rural doctors in this study. The multivariable logistic regression analyses were performed on the factors that may influence mental health via univariate analysis, the SCL-90 total score, and the nine-dimensional indicators used to evaluate mental health. The results are presented as an adjusted odds ratio (ORadj) with a 95% confidence interval (CI). A two-sided *P* value <0.05 was considered statistically significant.

### Ethics approval and consent to participate

This study was approved by the Medical Ethics Committee of **XX** (reference number: 2021089). All methods were performed in accordance with ‘Ethical Review Measures for Biomedical Research Involving Human Subjects’ and ‘Regulations of the Ethics Committee of **XX’**. Before filling out the questionnaire, the interviewees confirmed that they fully understood the precautions. Participation in the research was voluntary, and all participants provided informed consent.

## Results

The study distributed a total of 2665 questionnaires to different rural doctors in Hebei, and 2593 questionnaires were returned, resulting in a reasonable recovery rate of 97.30%. Among these 2593 rural doctors, 1872 (72.20%) were male. A total of 30.35% (787) of the participants were less than or equal to 40 years old, 40.99% (1063) were 41–50 years old, 14.69% (381) were 51–60 years old, and 13.96% (362) were over 60 years old. A total of 97.11% (2518) of the participants were married, 74.12% (1922) had lower than junior college degrees, 1.43% (37) had college degrees, and 98.42% of the participants had professional qualifications. Approximately 81.3% of rural doctors are in good health, 15.8% have chronic diseases, and 2.9% have disabilities. The average daily time spent on medical services was 11.82 hours, and 84.77% of rural doctors worked more than 8 hours a day.

A comparison of the total mean scores and factor scores obtained in this survey with the Chinese population norm revealed significant differences, except for SOM (P < 0.05). The total mean scores and factor scores are lower than the Chinese population norm, as shown in Table 1.

**Table 1.** Respondents’ scores in dimensions of SCL-90 (*n* = 2593)

Dimensions	Practical score (mean ± sd)	Chinese population norm (mean ± sd)	t	*P*
Overall	1.29 ± 0.454	1.44 ± 0.43	9.99	<0.001
SOM	1.34 ± 0.489	1.37 ± 0.48	1.83	0.067
OCS	1.45 ± 0.575	1.62 ± 0.58	8.76	<0.001
INTS	1.30 ± 0.510	1.65 ± 0.51	20.39	<0.001
DEPR	1.31 ± 0.515	1.50 ± 0.59	10.42	<0.001
ANX	1.26 ± 0.476	1.39 ± 0.43	8.38	<0.001
HOS	1.27 ± 0.507	1.48 ± 0.56	11.87	<0.001
PHOA	1.15 ± 0.401	1.23 ± 0.41	5.88	<0.001
PARI	1.22 ± 0.472	1.43 ± 0.57	12.29	<0.001
PSY	1.20 ± 0.438	1.29 ± 0.42	6.19	<0.001

* Chinese population norm: The Chinese population norms were derived from a representative state-wide sample (*N* = 1890) of Chinese adult residents in 2006 (Dai et al., 2015; Dong, 2010).

**Figure 2.**
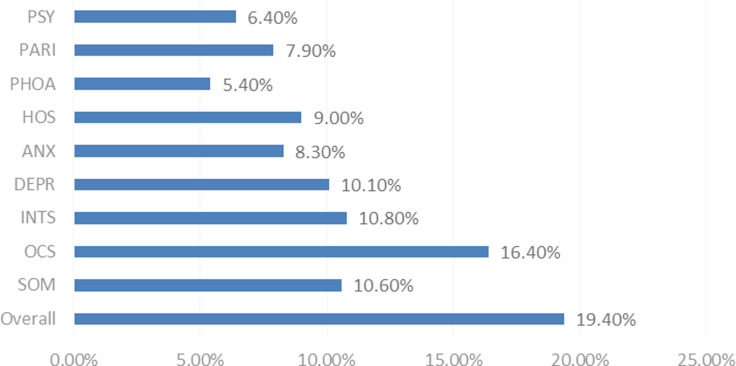
Detection rate in each dimension of SCL-90 among the rural doctors.

According to the screening principle of the SCL-90, the total positive rate of rural doctors was 19.40%. The detection rates of the first three dimensions were OCS (16.40%), INTS (10.80%), and SOM (10.60%). We also found that 10.10% of the responders might have been exposed to depression, 9.00% might have been exposed to hostility, 8.30% might have been exposed to anxiety, 7.90% might have been exposed to paranoid, 6.40% might have been exposed to phobic psychoticism, and 5.40% might have been exposed to phobic anxiety, See Figure 2.

The results of the analysis revealed differences in the psychological abnormalities of rural doctors of different ages, genders, health statuses, and working hours. The rates of abnormal total mean scores and the incidence of obsessive-compulsive disorder among rural doctors of different ages were statistically significant (*P* < 0.05). There were significant differences in the total mean scores of the rural doctors and the psychological abnormalities associated with the nine factors of sex, working hours, and health status. For significance (*P* < 0.05), see Table 2.

**Table 2.** Different characteristics of rural doctors SCL-90 abnormal rate [n(%)]

Influence factor	Number	Number of psychological abnormalities									
		Overall^1^^,^^2^^,^^3^^,^^4^	SOM^2^^,^^3^^,^^4^	OCS^1^^,^^2^^,^^3^^,^^4^	INTS^2^^,^^3^^,^^4^	DEPR^2^^,^^3^^,^^4^	ANX^2^^,^^3^^,^^4^	HOS^2^^,^^3^^,^^4^	PHOA^2^^,^^3^^,^^4^	PARI^2^^,^^3^^,^^4^	PSY^2^^,^^3^^,^^4^
Age											
≤40	787	179(22.7)	90(11.4)	159(20.2)	93(11.8)	84(10.7)	69(8.8)	75(9.5)	35(4.4)	68(8.6)	49(6.2)
41–50	1063	183(17.2)	96(9.0)	156(14.7)	115(10.8)	104(9.8)	81(7.6)	89(8.4)	60(5.6)	81(7.6)	65(6.1)
51–60	381	77(20.2)	46(12.1)	57(15.0)	40(10.5)	37(9.7)	34(8.9)	36(9.4)	18(4.7)	28(7.3)	26(6.8)
≥61	362	64(17.7)	43(11.9)	52(14.4)	32(8.8)	38(10.5)	30(8.3)	34(9.4)	27(7.5)	29(8.0)	27(7.5)
Gender											
Male	1872	382(20.4)	214(11.4)	328(17.5)	231(12.3)	213(11.4)	175(9.4)	189(10.1)	115(6.1)	171(9.1)	140(7.5)
Female	721	121(16.8)	61(8.5)	96(13.3)	49(6.8)	50(6.9)	39(5.4)	45(6.2)	25(3.5)	35(4.9)	27(3.7)
Marital status											
Spinsterhood	12	2(16.7)	2(16.7)	2(16.7)	1(8.3)	2(16.7)	2(16.7)	2(16.7)	0(0.0)	1(8.3)	1(8.3)
Married	2518	488(19.8)	267(10.6)	410(16.3)	277(11.0)	255(10.1)	211(8.4)	229(9.1)	139(5.5)	204(8.1)	164(6.5)
Dissociation	25	4(16.0)	3(12.0)	5(20.0)	0(0.0)	2(8.0)	0(0.0)	0(0.0)	0(0.0)	0(0.0)	0(0.0)
Widowed	38	9(23.7)	3(7. 9)	7(18.4)	2(5.3)	4(10.5)	1(2.6)	3(7.9)	1(2.6)	1(2.6)	2(5.3)
Practicing requirements											
Not have	41	9(22.0)	4(9.8)	9(22.0)	7(17.1)	6(14.6)	6(14.6)	5(12.2)	1(2.4)	6(14.6)	4(9.8)
Country doctor	1707	312(18.3	179(10.5)	261(15.3)	177(10.4)	158(9.3)	132(7.7)	143(8.4)	99(5.8)	129(7.6)	107(6.3)
Practicing assistant physician	561	120(21.4)	59(10.5)	103(18.4)	61(10.9)	66(11.8)	49(8.7)	52(9.3)	25(4.5)	44(7.8)	32(5.7)
Medical practitioner	284	62(21.8)	33(11.6)	51(18.0)	35(12.3)	33(11.6)	27(9.5)	34(12.0)	15(5.3)	27(9.5)	24(8.5)
Education											
Not have	104	18(17.3)	8(7.7)	13(12.5)	8(7.7)	7(6.7)	5(4.8)	10(9.6)	5(4.8)	6(5.8)	6(5.8)
Technical secondary school	1818	340(18.7)	188(10.3)	291(16.0)	189(10.4)	185(10.2)	149(8.2)	158(8.7)	100(5.5)	140(7.7)	118(6.5)
Junior college	634	137(21.6)	73(11.5)	113(17.8)	78(12.3)	67(10.6)	57(9.0)	61(9.6)	33(5.2)	57(9.0)	40(6.3)
Undergraduate	37	8(21.6)	6(16.2)	7(18.9)	5(13.5)	4(10.8)	3(8.1)	5(13.5)	2(5.4)	3(8.1)	3(8.1)
Health situation											
Disability	75	22(29.3)	13(17.3)	20(26.7)	12(16.0)	9(12.0)	11(14.7)	12(16.0)	5(6.7)	10(13.3)	7(9.3)
Chronic	409	135(33.0)	98(24.0)	114(27.9)	73(17.9)	80(19.6)	66(16.1)	71(17.4)	50(12.2)	62(15.2)	59(14.4)
Good	2109	346(16.4)	164(7.8)	290(13.8)	195(9.3)	174(8.3)	137(6.5)	151(7.2)	85(4.0)	134(6.4)	101(4.8)
Working time											
1–3	126	25(13.2)	12(6.3)	25(13.2)	17(9.0)	15(7.9)	11(14.7)	15(7.9)	10(5.3)	15(7.9)	10(5.3)
4–7	269	37(8.1)	27(5.9)	28(6.1)	18(3.9)	20(4.4)	15(3.3)	18(3.9)	9(2.0)	15(3.3)	14(3.1)
8–11	853	134(11.9)	68(6.0)	106(9.4)	63(5.6)	59(5.2)	52(4.6)	58(5.1)	33(2.9)	47(4.2)	34(3.0)
≥12	1345	307(37.7)	168(20.6)	265(32.5)	182(22.3)	169(20.7)	136(16.7)	143(17.6)	140(17.2)	129(15.8)	109(13.4)

* Chi-square test is used for comparison between groups, *P* < 0.05.

1 represents the comparison of rural doctors of different ages, *P* < 0.05

2 means the comparison of rural doctors of different genders, *P* < 0.05

3 represents the comparison of rural doctors with different health status, *P* < 0.05

4 represents the comparison of rural doctors with different working hours, *P* < 0.05.

** In education, ‘No have’ means that there is no medical degree.

Table 3 shows that age, gender, education, health status, and working time are the main factors influencing the mental health of rural doctors, with gender, age, health status, and working time being particularly significant. The risk of mental health problems among rural female doctors was lower than that among male doctors (OR < 1). Rural doctors with chronic diseases or disabilities were at greater risk of mental health problems. The risks of horror and psychotic symptoms were very high (OR > 3). Younger rural doctors and those with longer working experience were more likely to experience mental health problems. Additionally, rural doctors with higher education levels had a greater risk of somatization (OR = 1.371).

**Table 3.** Results of multivariate logistic regression analysis

		B	Wald	*P*	OR	OR 95% CI	
						Lower	Upper
Overall							
	Age	−0.233	18.585	<0.001	0.792	0.712	0.880
	Gender	−0.237	3.992	0.046	0.789	0.626	0.996
	Health situation						
	Good						
	Disability	0.803	9.274	0.002	2.233	1.331	3.743
	Chronic	1.073	68.613	<0.000	2.923	2.268	3.768
	Working time	0.185	8.274	0.004	1.203	1.061	1.364
SOM							
	Education	0.316	7.076	0.008	1.371	1.087	1.730
	Health situation						
	Good						
	Disability	0.965	9.273	0.002	2.626	1.411	4.888
	Chronic	1.370	91.171	<0.001	3.935	2.970	5.213
OCS							
	Age	−0.297	25.717	<0.001	0.743	0.663	0.834
	Gender	−0.343	7.054	0.008	0.710	0.551	0.914
	Health situation						
	Good						
	Disability	0.896	10.774	0.001	2.451	1.435	4.186
	Chronic	1.076	61.600	<0.001	2.934	2.243	3.839
	Working time	0.177	6.599	0.010	1.194	1.043	1.366
INTS							
	Age	−0.225	10.914	0.001	0.799	0.699	0.913
	Gender	−0.671	16.181	<0.001	0.511	0.369	0.709
	Health situation						
	Good						
	Disability	0.623	3.615	0.057	1.865	0.981	3.545
	Chronic	0.856	28.882	<0.001	2.355	1.723	3.218
	Working time	0.215	6.626	0.010	1.240	1.053	1.460
DEPR							
	Age	−0.161	5.419	0.020	0.851	0.744	0.975
	Gender	−0.518	9.616	0.002	0.596	0.429	0.826
	Health situation						
	Good						
	Disability	0.401	1.195	0.274	1.493	0.728	3.065
	Chronic	1.060	45.179	<0.001	2.888	2.120	3.934
	Working time	0.194	5.180	0.023	1.214	1.027	1.436
ANX							
	Age	−0.170	5.036	0.025	0.844	0.728	0.979
	Gender	−0.553	8.797	0.003	0.575	0.399	0.829
	Health situation						
	Good						
	Disability	0.894	6.843	0.009	2.444	1.251	4.774
	Chronic	1.087	40.055	<0.001	2.964	2.117	4.150
	Working time	0.204	4.666	0.031	1.227	1.019	1.477
HOS							
	Age	−0.170	5.549	0.018	0.844	0.733	0.972
	Gender	−0.487	7.712	0.005	0.614	0.435	0.866
	Health situation						
	Good						
	Disability	0.917	7.778	0.005	2.501	1.313	4.765
	Chronic	1.090	43.287	<0.001	2.973	2.149	4.113
PHOA							
	Age	−0.493	4.707	0.030	0.611	0.391	0.953
	Health situation						
	Good						
	Disability	0.472	0.978	0.323	1.603	0.629	4.081
	Chronic	1.155	37.690	<0.001	3.175	2.196	4.591
PARI							
	Age	−0.213	7.672	0.006	0.808	0.696	0.940
	Gender	−0.663	11.645	0.001	0.515	0.352	0.754
	Health situation						
	Good						
	Disability	0.831	5.503	0.019	2.297	1.147	4.600
	Chronic	1.075	37.683	<0.001	2.929	2.078	4.129
PSY							
	Gender	−0.625	8.283	0.004	0.535	0.350	0.819
	Health situation						
	Good						
	Disability	0.644	2.458	0.117	1.905	0.851	4.262
	Chronic	1.157	43.851	0.000	3.181	2.259	4.480

## Discussion

### Findings and interpretation

1. **Mental health status of rural doctors in Hebei Province**

According to our survey, rural doctors in Hebei Province have better psychological conditions than those in other provinces do (Wu, 2011; Lin and Li, 2016; Tian *et al*., 2021). First, our investigation was conducted recently, which could be a reason for the results. With the development of medical technology, medical reform policies, and increasing emphasis on primary medical care and rural doctors, the mental health problems of rural doctors have decreased. Second, the findings may be influenced by survey locations. Because different regions have different support policies, Hebei Province is committed to strengthening the benefits of rural doctors, conscientiously implementing and improving policies for elderly individuals, and strengthening subsidies for public health services in township hospitals (Zhu, 2021), which also affects the results. Compared with doctors in some general hospitals in China, rural doctors have better psychological conditions, and their health levels are lower than the Chinese population norm (Gu *et al*., 2008; Zou *et al*., 2015; Lu, 2015). On the one hand, rural doctors are more responsible for treating minor diseases, such as colds, with less difficulty and less pressure than doctors in higher-level hospitals are. On the other hand, currently, patients are more inclined to visit higher-level hospitals, and most of the initial doctors are doctors at the county level and above hospitals. Compared with doctors in higher-level hospitals, rural doctors have fewer consultations and shorter working hours. Therefore, rural doctors have relatively better mental health.

Although the survey results revealed that the mental health of rural doctors in Hebei Province is better than the Chinese population norm, 19.4% still screened positive on the scale. Compared with foreign studies, Chinese rural doctors have more unsatisfactory psychological conditions (Pappas *et al*., 2016), so they still need to pay attention. Rural doctors are sequelae of China’s health service development, and they are in a relatively low position. Most of them have no formal professional school education, which leads to a certain degree of professional inferiority complex (Ren, 2007), relatively low wages, and social status. They should be called farmers rather than doctors, but they must undertake heavy rural medical and health services. However, doctors or family doctors have higher social status and better incomes abroad, which is the main reason for the difference in mental health.

1. **Factors influencing the psychological condition of rural doctors**

In our study, we found that five factors affected the mental state of rural doctors. The first influencer is age. The younger the rural doctors are, the greater the risk of mental health problems. Young rural doctors, who are just starting to work lack clinical and social experience, are under high pressure for promotion and life and are prone to mental health problems (Sun and Zhang, 2014; Wu *et al*., 2019). In addition, the ‘Suspected Survival and Working Status Report in the Context of the New Medical Reform’ issued by the Chinese Academy of Social Sciences in 2012 stated, ‘After the implementation of the zero markups, the income of clinics dropped by 98.2%. The village clinics were operated by themselves in the past, and the main income was added drugs. After the zero markups, it is difficult for them to maintain operation’ (Han *et al*., 2014). Therefore, as the income of village clinics decreases, the income of young village doctors decreases, resulting in dissatisfaction and poor mental health. Moreover, in China, the profession of rural doctors is a family-oriented process that is jointly built by multiple generations (Gao, 2018). Young rural doctors must consider their development and professional ambitions and factors such as the reputation of their predecessors and their self-satisfaction with their careers, which will also affect their mental health.

The second influencing factor is gender. The mental health risk of rural male doctors is greater than that of female doctors. First, most rural doctors in China are men. They are the primary source of family income and are under pressure from various aspects of society and family; therefore, they are more likely to experience unhealthy mental conditions such as fatigue and depression, and to a certain extent, these conditions also lead to a decrease in work concentration and motivation. The sleep state of rural doctors is also affected, resulting in a decrease in work efficiency, which is not conducive to the treatment of patients (Wang and Gao, 2015). Men take on more social roles, have a stronger sense of work responsibility, and bear higher societal expectations and requirements, increasing work pressure on male doctors (Dai, 2012). Second, men have higher expectations of all aspects of work than women do, and it is difficult for rural doctors to mobilize their enthusiasm for work (Han *et al*., 2014). In addition, male doctors tend to have a tougher work attitude, whereas female doctors are relatively better at communication, which makes patients more willing to accept and obey doctors’ orders and results in fewer doctor-patient conflicts.

Third, the longer medical services are provided, the greater the risk of mental health problems for doctors. In China’s basic public health tasks, on average, each rural doctor has to establish files for 1328 people and, at the same time, be responsible for basic medical services (Wang, 2012). However, the number of rural doctors is far below the requirements set by the state. It is common for Chinese doctors to work at least 60 hours a week or take long shifts (Dai *et al*., 2015). Many doctors are busy and stressed. There is much evidence that there is a positive correlation between long working hours and mental health problems (Richter *et al*., 2014; de Cordova *et al*., 2014; Bannai and Tamakoshi, 2014). Owing to the shortage of human resources, doctors often have to work overtime or even insist on working with illness. Long working hours and heavy workloads can easily cause psychological problems (Hao, 2015). In addition, because rural doctors provide medical services for too long, they will have more physical expenditure and emotional investment, which can easily lead to endocrine hormone disorders, irritability, anxiety, and physical and mental exhaustion, leading to mental health problems.

Physical health is the fourth influencing factor. Rural doctors with chronic diseases or disabilities are at greater risk of psychological problems. We find that mental (physical) health has a significant direct and indirect impact on physical (mental) health. The indirect effect of mental health on physical health is mediated by lifestyle choices and social interactions. The relationship between physical health and mental health is mediated only by past physical activity (Ohrnberger *et al*., 2017). Rural doctors have heavy workloads, long working hours, frequent night visits, and irregular lives, making them feel exhausted and seriously affecting their physical and mental health (Lin and Li, 2016). The synergy between physical disability or illness and excessive medical service hours also increases mental health problems for rural doctors (Xu *et al*., 2013). In addition, drugs used to treat mental illness often cause side effects such as rapid weight gain and sleep disorders, resulting in impaired physical health (Happell *et al*., 2017). Although the work pressure of rural doctors is not as high as that of specialist doctors, they are still under much pressure because of their long working hours and trivial work content, which may further aggravate their psychological problems, thus forming a vicious cycle.

Fifth, rural doctors with a high degree of education are at greater risk of psychological problems. First, doctors with high academic qualifications have a lower sense of accomplishment than doctors with low academic qualifications (Lu *et al*., 2014), and their professional satisfaction is correspondingly lower. Being in this mood for a long time can induce psychological problems. Second, rural doctors with higher education levels have a more comprehensive range of knowledge and more careful consideration of things, and they are accustomed to suppressing their subconscious desires, which tends to cause psychological problems over time (Tong *et al*., 2018). In addition, rural doctors with higher education levels and more potent professional abilities are highly recognized by villagers (Gao, 2018), which results in longer working hours, greater intensity, and greater psychological pressure.

1. **Suggestions for improving the mental health of rural doctors**

As its economy has developed and the level of its health system has improved, China is constantly upgrading the level of rural doctors through medical education, optimizing the structure of rural doctors, broadening the space for their career development, compensating them through multiple channels and increasing their income levels so that they can better serve China’s primary health care (Meng and Yuan, 2023). However, the quality of life of rural doctors still has to be continuously improved, and their psychological problems must be taken seriously. Rural doctors should be given a certain degree of preference in terms of policy. First, support for rural doctors should continue to increase in the following areas: the first is to train suitable talent following the actual situation; the second is to make reasonable compensation from the economic point of view and improve salaries and wages; and the third is to continuously adjust the planning of talent training by development. Moreover, free regular medical check-up treatment should be provided for physical health-related illnesses so that rural doctors can keep abreast of their health status, prevent chronic diseases and other illnesses from occurring, and form a virtuous cycle of physical-mental health. Additionally, mental health education and psychological counselling lectures should be carried out regularly to assist rural doctors in preventing and solving their psychological problems. In addition, it is necessary to recognize rural doctors at the social level to increase their professional identity and sense of belonging. They support their families and provide good support for rural doctors. To reduce the incidence of their psychological problems, their capacity to provide primary health care services should be improved, and better primary health care services should be provided to the general population.

### Strengths and weaknesses

Hebei Province, with 0.2% of China’s area, nurtures 5.28% of the country’s population and is a veritable populous province, of which 56.4% are farmers. This study conducted a survey of rural doctors in several counties in 11 cities of Hebei Province. The scale of the survey was larger and more representative than those used in previous studies. Second, the survey objectively judges the mental health of rural doctors by comparing the results of the SCL-90 with the Chinese population norm and determines the influencing factors of the mental health of rural doctors from different aspects of personal conditions. These findings will contribute to improving the mental health of rural doctors and enable them to provide better primary health care services to the population at large.

The first limitation of this study is that the norm used for comparison dates back to 2006, which may not fully reflect the psychological status of the Chinese population at the time of the survey; however, the lack of recent survey results for comparison affects our comparison results. Additionally, as China lacks established mental health standards for urban doctors, our study omitted comparisons between urban and rural doctors. The second limitation of our study was the reliance on self-reports of SCL-90 sensitive items, such as thoughts of hurting others. Although anonymous self-report questionnaires were used, they might be vulnerable to social expectation bias.

## Conclusion

Compared with the Chinese population norm, rural doctors in Hebei Province normally exhibit good mental health; however, 19.4% still experience psychological problems. Young male rural doctors, those with longer medical service durations, chronic illnesses or disabilities, and higher levels of education are at greater risk of developing mental health problems. Attention should be given to the mental health of the rural doctor population to improve primary health care services.

## Data Availability

The datasets used and/or analysed in the present study are available from the corresponding author upon reasonable request.

## References

[ref1] Bannai A and Tamakoshi A (2014) The association between long working hours and health: a systematic review of epidemiological evidence. Scandinavian Journal of Work, Environment & Health 40(1), 5–18. 10.5271/sjweh.3388 24100465

[ref2] Bech P , Bille J , Moller SB , Hellström LC and Ostergaard SD (2014) Psychometric validation of the Hopkins Symptom Checklist (SCL-90) subscales for depression, anxiety, and interpersonal sensitivity. Journal of Affective Disorders 160, 98–103. 24445132 10.1016/j.jad.2013.12.005

[ref3] Chen Q , Wang B and Qu Y (2009) Investigation on Mental Health of Grass Roots Medical Staffs in Gansu Province. China Journal of Health Psychology 17, 1326–1328.

[ref4] Chen Y , Li X , Pan B , Liu Q , Ye J , Ge X , Yang K and Han X (2019) Basic medical and health services of doctors in the rural area under the background of hierarchical medical system. Chinese Rural Health Service Administration 39, 736–741.

[ref5] Dai S (2012) Analysis of SCL-90 test results of doctors in primary hospitals of Xichang City. Journal of Sichuan Minzu College 21, 105–108.

[ref6] Dai Y , Zhang B , Sun H , Li Z , Shen L and Liu Y (2015) Prevalence and correlates of psychological symptoms in Chinese doctors as measured with the SCL-90-R: a meta-analysis. Research in Nursing & Health 38, 369–383. 26291179 10.1002/nur.21673

[ref7] de Cordova PB , Phibbs CS , Schmitt SK and Stone PW (2014) Night and day in the VA: associations between night shift staffing, nurse workforce characteristics, and length of stay. Research in Nursing & Health 37(2), 90–97. 24403000 10.1002/nur.21582 PMC3959218

[ref8] Derogatis LR , Rickels K and Rock AF (1976) The SCL-90 and the MMPI: a step in the validation of a new self-report scale. British Journal of Psychiatry 128, 280–289. 10.1192/bjp.128.3.280 1252693

[ref9] Dong H (2010) A research of twenty years’ Vicissitude: SCL-90 and its norm. Journal of Psychological Science 33, 928–930.

[ref10] Dore I and Caron J (2017) Mental health: concepts, measures, determinants. Sante Mentale au Quebec 42, 125–145. 28792565

[ref11] Gao D (2018) Collapse of reproduction mechanism and social exclusion: research on “Brain Drain” of doctors in rural China. Journal of Sichuan Administration College 6, 70–76.

[ref12] Garelick AI , Gross SR , Richardson I , von der Tann M , Bland J and Hale R (2007) Which doctors and with what problems contact a specialist service for doctors? A cross sectional investigation. BMC Medicine 5, 26. 17725835 10.1186/1741-7015-5-26 PMC2025601

[ref13] Gazelle G , Liebschutz JM and Riess H (2015) Physician burnout: coaching a way out. Journal of General Internal Medicine 30, 508–513. 25527340 10.1007/s11606-014-3144-y PMC4371007

[ref14] Gu M , Gu Y , Mei Y , Lu J and Yu R (2008) Survey on mental health status of medical practitioners in comprehensive hospitals of Jiangsu. Chinese Journal of Public Health 8, 921–922.

[ref15] Han X , Wang L , Liang Y , Hu Q , Shao Y , Wang Z , Han Y and Chen Y (2014) Analysis of village doctors’ job satisfaction in Lanzhou City based on structural equation. Chinese General Practice 17, 2933–2936.

[ref16] Hao Y (2015) New year’s new thinking-pay attention to the physical and mental health of medical staff. Chinese Journal of Hypertension 23, 106–108.

[ref17] Happell B , Wilson K , Platania-Phung C and Stanton R (2017) Physical health and mental illness: listening to the voice of carers. Journal of Mental Health 26, 127–133. 27102585 10.3109/09638237.2016.1167854

[ref18] Harvey SB , Epstein RM , Glozier N , Petrie K , Strudwick J , Gayed A , Dean K and Henderson M (2021) Mental illness and suicide among physicians. Lancet 398, 920–930. 34481571 10.1016/S0140-6736(21)01596-8 PMC9618683

[ref19] Hu D , Zhu W , Fu Y , Zhang M , Zhao Y , Hanson K , Martinez-Alvarez M and Liu X (2017) Development of village doctors in China: financial compensation and health system support. International Journal for Equity in Health 16, 9. 28666444 10.1186/s12939-016-0505-7 PMC5493879

[ref20] Liang H and Lv R (2019) Analysis on the dilemma and path of collective economic development under the background of rural revitalization strategy. Bridge of Century 83–85.

[ref21] Lin H , Wang X , Hu X , Zhang X , Zhai J and Cai W (2017) Analysis on relationship of chronic fatigue and mental health of medical staff in Zhuhai City. Industrial Health and Occupational Diseases 43, 140–144.

[ref22] Lin W , Deng K , Xie X and Pan S (2013) Investigation on the mental health status of medical staff in the top three hospitals in Nanchang. Jiangxi Medical Journal 48, 151–154.

[ref23] Lin Y and Li P (2016) Research on the mental health status of rural doctors and the source of work stress. Psychologist 22, 229.

[ref24] Lin Y , Wei Y and Zhang J (2014) Research on the mental health status of rural doctors. China Health Care Nutrition 7, 4040–4041.

[ref25] Liu Y (2004) China’s public health-care system: facing the challenges. Bulletin of the World Health Organization 82, 532–538. 15500285 PMC2622899

[ref26] Lu J (2015) Investigation and analysis of mental health of medical staff in Jiangyin City. Sichuan Mental Health 28, 252–256.

[ref27] Lu M , Yan H , Zou T , Li L , Xue G and Wang Y (2014) The analysis of mental health and occupational burnout of community physicians. China Journal of Health Psychology 22, 542–545.

[ref28] Lv H , Yang C and Tian G (2018) Survey of mental health status of medical staff in Provincial General Hospital. Chinese Journal of Social Medicine 35, 379–381.

[ref29] Manwell LA , Barbic SP , Roberts K , Durisko Z , Lee C , Ware E and McKenzie K (2015) What is mental health? Evidence towards a new definition from a mixed methods multidisciplinary international survey. BMJ Open 5, e007079. 10.1136/bmjopen-2014-007079 PMC4458606 26038353

[ref30] Meng Q and Yuan B (2023) China’s experience in building a primary health care system and its significance for international reference. China Health Policy Research 16, 70–75.

[ref31] Ohrnberger J , Fichera E and Sutton M (2017) The relationship between physical and mental health: a mediation analysis. Social Science & Medicine 195, 42–49. 29132081 10.1016/j.socscimed.2017.11.008

[ref32] Pappas P , Gouva M , Gourgoulianis K , Hatzoglou C and Kotrotsiou E (2016) Psychological profile of Greek doctors: differences among five specialties. Psychology Health & Medicine 21, 439–447. 10.1080/13548506.2015.1090614 26399373

[ref33] Pattani S , Constantinovici N and Williams S (2001) Who retires early from the NHS because of ill health and what does it cost? A national cross sectional study. BMJ-British Medical Journal 322, 208–209. 10.1136/bmj.322.7280.208 PMC26586 11159617

[ref34] Ren R (2007) Heavy expectations: seven outstanding problems in the development of rural doctors in China. China Health Human Resources 000, 18–20.

[ref35] Richter A , Kostova P , Baur X and Wegner R (2014) Less work: more burnout? A comparison of working conditions and the risk of burnout by German physicians before and after the implementation of the EU Working Time Directive. International Archives of Occupational and Environmental Health 87, 205–215. 23423279 10.1007/s00420-013-0849-x

[ref36] Sun H and Zhang M (2014) A research report on the evaluation results of the Symptom Self-Rating Scale (SCL-90)—Study on the psychological evaluation results of part of the employees in the First People’s Hospital of Heihe City. Heihe Journal 6, 141–144.

[ref37] Tian Z , Lu J , Yang Y , Wang Y , Wang C and Zhen J (2021) Status quo and influencing factors of rural doctors’ mental health in Shanxi Province. Chinese Rural Health Service Administration 41, 543–548.

[ref38] Tong Y , Jiang ZQ , Zhang YX , Jia JL , Lu W , Wang J , Tang H , Zhang M , Guo XM , Li T , Jiang HY , Yu WL and Lou JL (2018) Analyzing the mental health status and its impact factors among female nurses in China. Zhonghua Lao Dong Wei Sheng Zhi Ye Bing Za Zhi 36, 115–118. 29699010 10.3760/cma.j.issn.1001-9391.2018.02.009

[ref39] Wang G and Gao H (2015) Investigation and research on the mental health of rural doctors. Modern Regimen 24, 232.

[ref40] Wang G and Wang Y (2014) Research on the psychological problems of rural doctors in the reform of rural health system in Jilin Province. Journal of Jilin Medical College 35, 441–443.

[ref41] Wang X (2012) Rural doctors are in an unprecedented dilemma. Chinese Community Physician 28, 3.

[ref42] Wang X , Hua L and Wang J (2013) Analysis on mental health status of 2460 health care workers in Beijing. Chinese Journal of Health Education 29, 779–781.

[ref43] Wang Z (1984) Symptom Self-rating Scale (SCL-90). Shanghai Archives of Psychiatry 2, 68–70.

[ref44] Wu H , Zhao Y , Wang JN and Wang L (2010) Factors associated with occupational stress among Chinese doctors: a cross-sectional survey. International Archives of Occupational and Environmental Health 83, 155–164. 19701645 10.1007/s00420-009-0456-z

[ref45] Wu J (2011) Investigation on mental health status of rural doctors in Jiangxi province. Health Vocational Education 29, 104–105.

[ref46] Wu Y , Wang L and Ren L (2019) Job burnout of rural doctors in Jilin province. Chinese Rural Health Service Administration 39, 57–61.

[ref47] Xu L , Xu Z and Xu S (2013) Research on the physical and mental status and its related factors of the hospital medical staff in Hubei Province. Chinese Journal of Social Medicine 30, 104–106.

[ref48] Zhang Q , Chen J , Yang M , Pan J , Li X , Yue L , Huang Y , Mao T , Zhang C and Ma X (2019) Current status and job satisfaction of village doctors in western China. Medicine 98, e16693. 31393371 10.1097/MD.0000000000016693 PMC6709036

[ref49] Zhang T and Yang T (2019) The tension of folk medicine practice: a survey of rural doctors in Yangqu Village, Jiaocheng County, Shanxi Province. Journal of Lanzhou Institute of Education 35, 81–83.

[ref50] Zhu S (2021) Chengde, Hebei: rural doctors can keep and serve. China Health 4, 44–45.

[ref51] Zou Z , Huang Y , Wang J , Li X , He Y , Min W and Zhou B (2015) The investigation of mental health status of medical staffs in a general hospital. Practical Journal of Clinical Medicine 12, 78–81.

